# Confirmation of warfarin resistance of naturally occurring VKORC1 variants by coexpression with coagulation factor IX and *in silico* protein modelling

**DOI:** 10.1186/1471-2156-15-17

**Published:** 2014-02-04

**Authors:** Elisabeth Müller, Alexander Keller, Andreas Fregin, Clemens R Müller, Simone Rost

**Affiliations:** 1 Department of Human Genetics, University of Würzburg, Würzburg, Germany; 2 DNA Analytics Core Facility, Biocenter, University of Würzburg, Würzburg, Germany; 3 Department of Animal Ecology and Tropical Biology, University of Würzburg, Würzburg, Germany

**Keywords:** VKORC1, Vitamin K epoxide reductase, Anticoagulants, Warfarin, Coumarin, Coexpression, Coagulation factor IX

## Abstract

**Background:**

VKORC1 has been identified some years ago as the gene encoding vitamin K epoxide reductase (VKOR) – the target protein for coumarin derivates like warfarin or phenprocoumon. Resistance against warfarin and other coumarin-type anticoagulants has been frequently reported over the last 50 years in rodents due to problems in pest control as well as in thrombophilic patients showing variable response to anticoagulant treatment. Many different mutations have already been detected in the VKORC1 gene leading to warfarin resistance in rats, mice and in humans. Since the conventional *in vitro* dithiothreitol (DTT)-driven VKOR enzymatic assay often did not reflect the *in vivo* status concerning warfarin resistance, we recently developed a cell culture-based method for coexpression of VKORC1 with coagulation factor IX and subsequent measurement of secreted FIX in order to test warfarin inhibition in wild-type and mutated VKORC1.

**Results:**

In the present study, we coexpressed wild-type factor IX with 12 different VKORC1 variants which were previously detected in warfarin resistant rats and mice. The results show that amino acid substitutions in VKORC1 maintain VKOR activity and are associated with warfarin resistance. When we projected *in silico* the amino acid substitutions onto the published three-dimensional model of the bacterial VKOR enzyme, the predicted effects matched well the catalytic mechanism proposed for the bacterial enzyme.

**Conclusions:**

The established cell-based system for coexpression of VKORC1 and factor IX uses FIX activity as an indicator of carboxylation efficiency. This system reflects the warfarin resistance status of VKORC1 mutations from anticoagulant resistant rodents more closely than the traditional DTT-driven enzyme assay. All mutations studied were also predicted to be involved in the reaction mechanism.

## Background

Vitamin K hydroquinone is an essential cofactor for the posttranslational modification of vitamin K-dependent (VKD) proteins by the endoplasmic membrane enzyme γ-glutamyl-carboxylase (GGCX) [1]. VKD proteins comprise clotting factors II, VII, IX and X, protein C, S and Z as well as osteocalcin, matrix Gla protein, growth arrest-specific protein (gas6) and four transmembrane proteins (PRGP1, PRGP2, TMG3, TMG4) [2,3]. For these proteins, γ-carboxylation is essential in order to attain their full biological activity state. During each carboxylation step, vitamin K hydroquinone is oxidised to vitamin K epoxide which is in turn reduced by vitamin K epoxide reductase (VKOR) [4]. The gene encoding vitamin K epoxide reductase complex subunit 1 (VKORC1) was identified in 2004 by two independent working groups [5,6].

VKOR activity can be effectively inhibited by coumarin derivatives such as warfarin or phenprocoumon. Inhibition of VKOR leads to the secretion of under-carboxylated VKD clotting factors and therefore to reduced blood coagulation [7]. Coumarin derivatives are in world-wide use for prevention and therapy of thromboembolic conditions in humans and in higher doses as rodenticides. Resistance in rats and mice has been reported first for warfarin in 1960 [8,9] and some years later even for more potent anticoagulants [10,11] leading to serious local problems in pest control. Warfarin resistance has also been demonstrated in patients suffering from thromboembolic diseases who failed to respond to oral anticoagulant treatment [12,13].

To date, many different point mutations have been discovered in the VKORC1 gene of warfarin-resistant rats and mice [14-16] as well as in humans [5,13,17]. In house mice (*Mus musculus domesticus*), *vkorc1* mutations have not solely occurred spontaneously and positively selected by anticoagulant rodenticides but can also originate from the less warfarin-sensitive *Mus spretus* by “adaptive introgressive hybridization” (interspecific mating followed by introgression and selection on these alleles) [18]. In rats, several *vkorc1* mutations could be confirmed as warfarin resistance-mediating by *in vivo* and *in vitro* tests so far, while some variants were interpreted as natural interspecies differences, e.g. between *Rattus norvegicus* and *Rattus rattus*[19].

Upon site-directed mutagenesis in a VKORC1-cDNA construct, only mutations at tyrosine-139 showed distinct resistance to warfarin in an *in vitro* DTT-driven enzymatic assay for VKOR activity [14]. Since this standard VKOR assay could not clearly demonstrate warfarin resistance for most VKORC1 mutations, a cell culture-based coexpression system was recently developed by our group [20]. In this system, a bicistronic vector harbouring human VKORC1 and coagulation factor IX (FIX) cDNAs is transfected into mammalian cells. In combination with the endogenous carboxylase activity of these cells, the concomitant expression of VKORC1 and FIX allows to study the effects of mutations in the VKORC1 gene on the activity of the clotting factor, the end product of γ-carboxylation, and its inhibition by warfarin. The results from this cell-based assay accurately reflect the warfarin resistance phenotypes for three human VKORC1 mutations identified in anticoagulant insensitive patients [20].

In the present study, the coexpression system was used to study the effect of 12 different VKORC1 variants detected in rats and mice on the production of clotting factor IX in HEK 293 cells and its sensitivity to warfarin inhibition. In contrast to the DTT-driven enzymatic *in vitro* assay, in the cell-based assay mutations other than those affecting position 139 of VKORC1 showed resistance against warfarin. This is in good agreement with the observation of *in vivo* resistance to warfarin in rodents carrying such mutations. An *in silico* 3D modelling of the 12 amino acid substitutions into mammalian VKORC1 (based on the recently published structure of the bacterial homolog [21]) confirmed the relevance of these amino acids which are substituted in warfarin-resistant rodents with regard to protein structure and binding of ligands like vitamin K, ubiquinone or coumarin derivatives.

## Methods

### Cloning and mutagenesis

The bicistronic vector for VKORC1 and FIX coexpression was constructed by cloning FIX cDNA [GenBank: NM_000133] into a pCEP4 vector (Invitrogen, Karlsruhe, Germany) using 5′ *Nhe* I and 3′ *Xho* I restriction sites and inserting VKORC1 cDNA [GenBank: NM_024006] together with an IRES sequence (internal ribosome entry site) downstream from the FIX cDNA using 5′ *Sfi* I and 3′ *Bgl* II sites.

Site-directed mutagenesis was performed using the QuikChange mutagenesis kit (Stratagene, Amsterdam, NL) following the manufacturer’s instructions. Mutagenic primer pairs were about 30 base pairs in length and flanked the desired mutation position (primer sequences available on request).

### Cell culture and protein expression

The bicistronic vector constructs were transfected into HEK 293 EBNA cells (Invitrogen, Karlsruhe, Germany) using FuGene HD (Roche) and transfected cells were treated with different concentrations of warfarin (0, 0.01, 0.03, 0.1 or 0.3 μM final conc.) as described by Fregin et al. [20]. Supernatants of cell cultures containing secreted FIX were harvested after culturing for approx. 70 hours and concentrated by ultrafiltration (10,000 MWCO; Vivaspin 6, Sartorius Stedim Biotech S.A., France) after a short centrifugation step to remove floating cells according to the manufacturer’s conditions. Concentrates were resuspended in 800 μL Owren Veronal buffer (Dade Behring, Siemens Healthcare Diagnostics, Germany). Whole protein amounts of aliquots of each concentrate were determined relative to a BSA standard curve using the DC Protein Assay kit (BioRad, Germany) according to the manufacturer’s instructions.

FIX activities of concentrated supernatants were determined after adding aliquots of 10 μl to factor-depleted standard plasma (Siemens Healthcare Diagnostics Inc., Germany) using an Electra 1400c coagulometer (Instrumentation Laboratory). Standard pooled blood donor plasma (Siemens Healthcare Diagnostics Inc., Germany) served as a control. The activity of standard plasma was set to 100% activity for FIX and sample activities were normalized to this value.

### 2D and 3D protein structure analysis of VKORC1 variants

The three-dimensional model of wild-type mammalian vitamin K epoxide reductase was constructed *in silico* by embedding the human VKORC1 protein sequence (NP_076869) in the 3D crystal structure of the bacterial homolog of vitamin K epoxide reductase from *Synechococcus sp.* (PDB-3KP9; [21]) using SWISS-MODEL for prediction [22] and 3Drefine for refinement and evaluation [23]. They were also compared with two-dimensional protein checks which were done by the GOR IV secondary structure prediction method [24]. Binding sites of warfarin and hydroquinone were predicted using VINA [25]. All 12 mutations were inserted into the human VKORC1 protein sequence since the rodents’ sequences (from *Rattus norvegicus* and *Mus musculus*) are identical to human VKORC1 at all these positions. Considering the complete VKORC1 protein, mouse and human protein sequences are 92% homologous (85% identical) whereas rat and human VKORC1 proteins share 91% homology (83% identity). Three-dimensional models of wild-type and mutated VKORC1 variants were displayed using UCSF Chimera [26].

## Results and discussion

**Figure 1:**
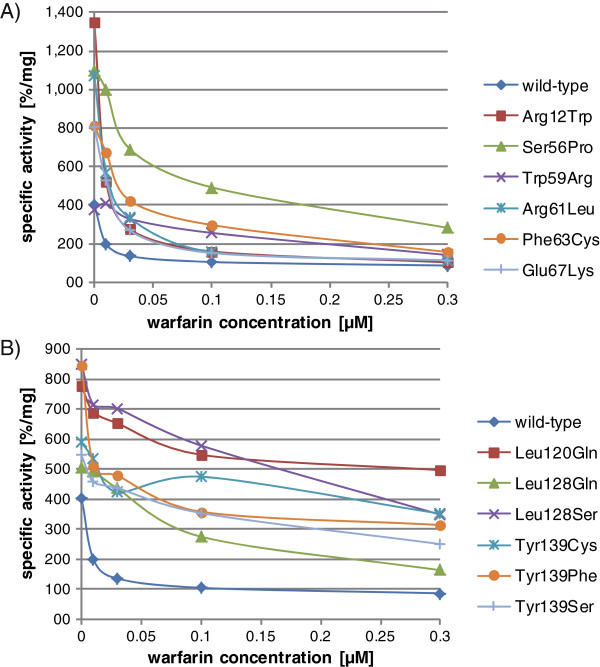
Specific activities of coagulation factor IX coexpressed with different VKORC1 variants: wild-type (= 100%) and (A): N-terminal variants at positions 12, 56, 59, 61, 63 and 67; (B): C-terminal variants at positions 120, 128 and 139 in the absence and presence of different warfarin concentrations.

Twelve VKORC1 variants which were detected in warfarin-resistant rats and mice in previous studies [14,15,18,19] were coexpressed with the coagulation factor FIX in mammalian cells (Table 1). Culture supernatants were collected and concentrated and FIX specific activities were determined based on the whole protein amounts of each sample assuming a linear relation of the amount of secreted FIX to the total protein content. For each variant, transfections and hence, FIX measurements were performed at least four times and mean values were calculated. Figure 1 shows the specific activities of FIX coexpressed with wild-type VKORC1 and the twelve VKORC1 variants, respectively. In the absence of warfarin, all variants showed approximately the same or even higher basal FIX activities compared to the wild-type and most variants appeared to be insensitive to all four warfarin concentrations tested. Three variants (Arg12Trp, Arg61Leu and Glu67Lys) showed specific activities only slightly higher than the wild-type in the presence of the two highest warfarin concentrations (0.1 and 0.3 μM final conc.). Two of these variants (Arg12Trp and Arg61Leu) were detected in *Mus musculus domesticus* and most likely originate from *Mus spretus* as described above. It is assumed that a genomic region of >10 Mb containing at least four of ten non-synonymous *vkorc1* SNPs was introgressed in the house mouse from *Mus spretus* conferring warfarin resistance [18]. Probably, each of these variants alone shows only minor warfarin resistance and only a combination of some of these variants causes distinct resistance. While in wild animals the genetic background may modulate the effect of *vkorc1* variants, for our *in vitro* studies we chose a homologous system (human VKORC1 cDNA in the human-derived cell line HEK 293) in order to study the variants independently from such epistatic effects.

**Table 1:** Overview of the 12 examined VKORC1 variants detected in mice and rats

**Mutation**	**Species/ref.**	**2D, 3D prediction**	**3D modelling**
Arg12Trp	*Mus musculus, Mus spretus*[15,18]	2D: tendency to random coil	
		3D: clash of side chain	
Ser56Pro	*Rattus norvegicus*[14]	2D: tendency to random coil	
		3D: clash of side chain, less hydrogen bonds	
Trp59Arg	*Rattus spec.*[15,19]	2D: tendency to random coil	
		3D: change of side chain for ligand	
Arg61Leu	*Mus musculus, Mus spretus*[15,18]	2D: tendency to beta strand	
		3D: no important differences	
Phe63Cys	*Rattus spec.*[15,19]	2D: tendency to beta strand	
		3D: clash of side chain	
Glu67Lys	*Rattus spec.*[15,19]	2D, 3D: no important differences	
Leu120Gln	*Rattus norvegicus*[14]	2D: conversion of helix to beta strand	
		3D: missing side chain for ligand binding	
Leu128Gln	*Rattus spec.*[14,15,19]	2D: partial conversion of helix to beta strand	
		3D: no differences, interaction with ligand?	
Leu128Ser	*Mus musculus*[14,15]	2D: partial conversion of helix to beta strand	
		3D: no differences, interaction with ligand?	
Tyr139Cys	*Rattus spec., Mus musculus*[14,15,19]	2D: tendency to beta strand	
		3D: no differences, interaction with ligand?	
Tyr139Phe	*Rattus spec.*[14,15,19]	2D, 3D: no differences, interaction with ligand?	
Tyr139Ser	*Rattus spec.*[14,15,19]	2D: tendency to random coil	
		3D: no differences, interaction with ligand?	

Results of two- and three-dimensional protein structure predictions are given in the third column. Pictures of 3D modelling based on human VKORC1 protein sequence and the bacterial VKOR structure show the protein parts comprising the respective substitutions. The wild-type protein is displayed in beige, the mutated protein is overlaid in light blue, side chain clashes are highlighted in red.

When comparing the results of the present study to the previous data of the DTT-driven VKOR assay [14,15], the cell culture-based coexpression data show distinct resistance against warfarin not only for VKORC1 variants at position 139 but also for variants Ser56Pro, Trp59Arg, Phe63Cys, Leu120Gln and those concerning Leu128. Some of these amino acid positions have not only been shown to be involved in warfarin resistance in rodents but are also found mutated in humans with elevated oral anticoagulant dosage requirements or complete resistance against coumarin-type anticoagulants: e.g. Ser56Phe (4-5x of normal anticoagulant dosage), Trp59Cys (3-4x of normal dosage), Trp59Leu (4-5x of normal dosage), Leu128Arg (4-5x of normal dosage) and Tyr139His (3-4x of normal dosage) [5,17]. Hence, the coexpression assay seems to reflect the *in vivo* observed ‘resistance conditions’ much more accurate than the DTT-driven VKOR assay, independently of the species in which those VKORC1 mutations were originally detected.

Three-dimensional modelling of all 12 VKORC1 mutations predicted side chain clashes for Arg12Trp, Ser56Pro and Phe63Cys. The strongest modifications of side chains for ligand binding are indicated for Trp59Arg and Leu120Gln while only small effects are predicted for Arg61Leu and Glu67Lys (Table 1). This is in good accordance with our experimental data (Figure 1A) in which the latter two variants show only slight insensitivity to warfarin.

**Figure 2:**
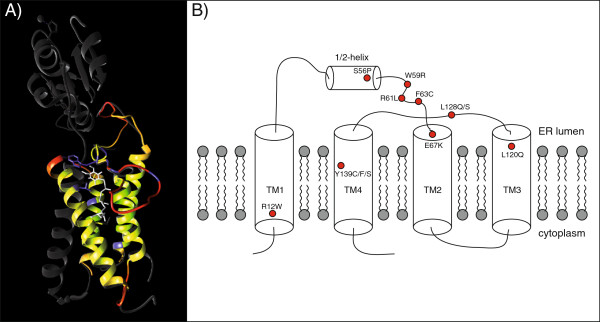
**Three-dimensional and topology models of the vitamin K epoxide reductase. A)***In silico* 3D model of human vitamin K epoxide reductase based on the homologous bacterial protein structure (PDB-3KP9; [21]). The four transmembrane helices of the core protein are displayed in yellow, protein structures which are only present in the bacterial homolog (Trx-like domain, linker and 5^th^ transmembrane domain) are shown in grey, imprecise regions in the human and bacterial protein structure are highlighted in red, positions of the 12 examined substitutions are represented in blue. The bound substrate (ubiquinone) is shown in white. The location of the interaction partner was verified for vitamin K1 within the human model using VINA [25] and found to be equivalent. **B)** Membrane topology model of the vitamin K epoxide reductase according to Li et al. [21] with the location of the 12 warfarin-resistant mutations examined in this study (red dots).

Our results support the idea of Rapoport’s working group [21] that the sensitivity of VKORC1 to inhibition by warfarin is not only mediated by the proposed TYA binding motif (Thr138-Tyr139-Ala140) which was previously studied by our working group using site-directed mutagenesis [27], but that other positions play an essential role as well. According to the published crystal structure of a bacterial VKOR homolog by Li et al. [21] it is most likely that the core of VKOR of all species (including those of mammals) consists of four transmembrane domains (TMs) bringing together on the luminal side of the ER membrane all amino acids which are important for enzyme activity. Figure 2 shows the VKORC1 mutations studied here projected into the *in silico* 3D model based on the bacterial VKOR structure (Figure 2A) and the modified VKOR membrane topology model with the essential four-helix bundle previously published by Li et al. (Figure 2B) [21]. The luminal loop between TM1 and TM2 contains a so-called ½ helix which forms a lid on the four-helix bundle. This “binding pocket” is large enough to embed one of the ligands: ubiquinone, vitamin K or coumarin derivatives like warfarin. Except for Arg12Trp, all eleven mutations of the present study map close to the binding site of the ligands as suggested by the 3D and topology models: Ser56 is located at the end of the ½ helix, Trp59, Arg61 and Phe63 immediately follow the ½ helix, Glu67 is located at the beginning of TM2, Leu120 at the end of TM3, Leu128 in the luminal loop between TM3 and TM4 and finally Tyr139 is part of the TYA warfarin binding motif in TM4. Mutations concerning amino acid residues 56-67 might affect the lid of the binding pocket and mutations 120-139 are supposed to lower the affinity for warfarin [21]. This hypothesis could explain the observed clustering of mutations in these two protein domains and the more pronounced warfarin resistance conferred by mutations in the C-terminal part of the protein (Figure 1B). Amino acids 120, 128 and 139 are presumably involved directly in binding of the inhibitor whereas amino acids in the N-terminal part are probably only required for directing the ligand into the binding pocket.

In 2012, Tie et al. published a model for the human vitamin K epoxide reductase with only three transmembrane domains (TMD) [28]. Their model is based on fluorescence protease protection assays after expression of VKOR fused to GFP (green fluorescence protein) which is a hydrophilic protein approximately twice the size of VKOR. Therefore, GFP when directly linked to VKOR may have an influence on membrane topology of its fusion partner. Tie at al. also reported that mutations in some charged VKOR residues can change the topology from three- to four-TMDs and that both variants are enzymatically active [28]. In performing our *in silico* 2D and 3D modelling of the human enzyme on basis of the bacterial homolog we also observed that both topologies are possible and are easily being converted into each other. *In silico*, a single substitution is sufficient to convert the three-TMD topology model into the four-TMD model and vice versa. This is further supported by the fact that membrane topology predictions of VKORs from different species using different prediction tools sometimes show the three- and sometimes the four-TMD topology depending on the software used. Upon crystallisation, the protein is “frozen” in a state of low energy requirement while modelling tools suggest that the native enzyme may be topologically more flexible. It would be conceivable that the membrane topology is either tissue-specific or that shifting between the three- and the four-TMD conformation of the vitamin K epoxide reductase is part of its reaction mechanism: the conformational transition could impact on opening and closing the lid of the binding pocket by a kind of flip-flop mechanism which could also be dependent on the ligand embedded in the pocket – either the substrate (vitamin K) or the inhibitor (warfarin).

## Conclusions

In conclusion, the cell-based FIX/VKORC1 coexpression assay measures VKOR activity indirectly via the activity of carboxylated FIX. This seems to reflect the physiological conditions of the vitamin K metabolism and its warfarin sensitivity more accurately than the conventional assay which measures VKOR activity directly in the presence of the non-physiological strong reductant DTT. Using this assay, we determined VKOR activities and warfarin sensitivities of 12 VKORC1 variants reported from acquired or naturally warfarin-resistant rodents. In contrast to the traditional enzyme assay, all variants displayed strong activities in the absence of anticoagulants and high to complete insensitivity towards warfarin. In addition, when these variants were modelled *in silico* onto the three-dimensional protein structure of VKOR from *Synechococcus sp.,* all but one variant affected protein domains suggested relevant for ligand binding by previous studies.

## Competing interests

The authors declare that they have no competing interests.

## Authors’ contributions

EM carried out all mutagenesis and expression experiments. AK performed *in silico* 2D and 3D protein structure analyses and revised the manuscript. AF performed cloning of the bicistronic expression vector and established the coexpression assay. CRM contributed substantially to the conception and design of the study and critically revised the manuscript. SR contributed to the interpretation of data, supervised the study and drafted the manuscript. All authors read and approved the final manuscript.
